# Simultaneous determination of six active metabolites in *Astragalus mongholicus* (Fisch.) Bge. under salt stress by ultra-pressure liquid chromatography with tandem mass spectrometry

**DOI:** 10.1186/s40064-016-2638-y

**Published:** 2016-06-30

**Authors:** Yang Liu, Jia Liu, Yu Wang, Ann Abozeid, Zhong-Hua Tang

**Affiliations:** Key Laboratory of Plant Ecology, Northeast Forestry University, Harbin, 150040 China; Botany Department, Faculty of Science, Menoufia University, Shebin El-koom, 32511 Egypt

**Keywords:** UPLC–MS, Bioactive metabolites, Salt stress, *Astragalus membranaceus Bge. var. mongholicus (Bge.) Hsiao*

## Abstract

*Astragalus membranaceus Bge. var. mongholicus (Bge.) Hsiao* (*A. mongholicus,* family Leguminosae) is one of the most important traditional Chinese herbs because it contains lots of bioactive metabolites, which have beneficial and pharmacological effects on health. Simultaneously, it has been proved to be a salt-tolerant plant—one of the potential species to control the soil salinization. Therefore, a sensitive and specific ultra-pressure liquid chromatography coupled with tandem mass spectrometric (UPLC-MS/MS) method was developed and validated for the simultaneous determination of six main bioactive metabolites, astragaloside IV, cycloastragenol, calycosin-7-*O*-β-d-glucoside, calycosin, ononin and formononetin in different organs of *A. mongholicus.* The detection was accomplished by multiple-reaction monitoring (MRM) scanning via electrospray ionization source operating in the positive ionization mode. Calibration curves offered linear ranges of two orders of magnitude with R^2^ > 0.99. The method was fully validated for the linearity, intra-day and inter day precisions, accuracy, recovery, matrix effect and stability. Then this method was successfully applied to detect the content of major bioactive metabolites in different plant organs of *A. mongholicus* under salt stress. Significant variations in the content of six bioactive metabolites were observed after been processed by different levels of salinity in different part of plant. The results support for further exploration of the salt-tolerant mechanisms in *A. mongholicus* and its possibility as the species that control the soil salinization. Meanwhile, we established a UPLC-MS/MS assay of the trace components in seedling of *A. mongholicus* in this study.

## Background

Radix Astragali, A widely used Chinese Herbal Medicine, is derived from the dried roots of *Astragalus mongholicus* (Fisch.) Bge. (Zu et al. 2009). *A. mongholicus* generally is mixed with other ingredients to make some medicated food in its edible aspect, which it has also traditionally been utilized in cosmetics. Pharmacological studies and clinical practice have demonstrated that *A. mongholicus* possess various biological activities including tonic, immunostimulant, hepatoprotective, diuretic, antidiabetic, cardioprotective, anti-oxidative and anti-tumor properties (Sun et al. 2008; Boye et al. 2015; Chen et al. 2015). More than 100 chemical constituents of *A. mongholicus* have been isolated and identified (Lv et al. 2015; Napolitano et al. 2013). Isoflavonoids and triterpene saponins have been considered as two types of the major bioactive metabolites found in *A. mongholicus.* Formononetin, ononin, calycosin and calycosin-7-*O*-β-d-glucoside, which boost energy, strengthen the immune system, and promote health activities and skin growth, are the major isoflavonoids in *A. mongholicus* (Xiao et al. 2004; Krasteva et al. 2015). Astrageloside IV has protective effects on cardiovascular system, immune, digestive and nervous system (Ren et al. 2013). Cycloastragenol is the synthetic precursor compound of astragaloside IV. And all of them could be the marker compounds for the chemical evaluation of *A. mongholicus* (Yesilada et al. 2005; Pu et al. 2015). In addition, formononetin, ononin, calycosin and calycosin-7-*O*-β-d-glucoside, are the most important metabolites in isoflavonoids biosynthesis pathway (Xu et al. 2011); astragaloside IV and cycloastragenol, are the essential metabolites of triterpene saponin biosynthesis pathway (Park et al. 2015). Synthesis or decomposition of these compounds has important implications for the quality of *A. mongholicus* as medicines (Zheng et al. 2015; Liu et al. 2015a).

Salinity is possibly the most imperative ecological restriction that causes extensive crop yield losses all over the world, and its threat is escalating day by day (Peng et al. 2011; AbdElgawad et al. 2016). The only way to control the soil salinization process and to maintain the sustainability of landscape and agricultural fields is to combat the salinization problems by environmentally safe and clean techniques, such as: use of salt-tolerant species (Hasanuzzaman et al. 2014; Moore 1984). *A. mongholicus* has been proved to be a salt-tolerant plant (Wdowiak-Wrobel et al. 2013), it was one of the potential species to control the soil salinization. So it is extremely critical to search for the salt-tolerance and the ability of physiological adaptation of *A. mongholicus.* When plants are exposed to salt stresses, they display intricate regulatory mechanisms to enhance their response, including cellular changes and metabolic responses (Molinier et al. 2006; Bruce et al. 2007; Chen et al. 2007). Importantly, we must focus on the content of major bioactive metabolites in *A. mongholicus.* under salt stress to ensure the medicinal value of *A. mongholicus*, then we can explore the possibility of *A. mongholicus* to be used as potential salinity species. As two types of the major metabolites and active components in *A. mongholicus,* the change in isoflavonoids and triterpene saponins level needs to be observed carefully. So we developed and applied the rapid and sensitive UPLC-MS method for simultaneous determination of astragaloside IV, cycloastragenol, calycosin-7-*O*-β-d-glucoside, calycosin, ononin and formononetin in *A. mongholicus* under different levels of salt stresses.

Because of the trace amounts of compounds in the seedlings, their quantitative analysis in the seedlings was difficult. In attempts to improve the determination of these compounds in *A. mongholicus*, several studies have been reported. The current method for the determination of these compounds mainly uses high-performance liquid chromatography (HPLC) coupled with DAD or ESI–MS Detection (Kwon and Park 2012; Gong et al. 2015). Being the common analytical tool for various compounds, HPLC and LC–MS both are being more and more widely applied in biological research, and have been used to quantify the marked compounds in biological samples such as *A. mongholicus* seedlings (Liu et al. 2015a; b). However, the threshold of sensitivities of these HPLC methods for detecting these compounds in *A. mongholicus* seedling is high. Furthermore, the major weakness of these LC–MS methods mainly includes chromatographic running time, which at more than 18 min is considered too long. This is time consuming and not suitable for analyzing large number of sample. Even though some experiments were investigated for determination of some compounds in *A. mongholicus* (Lv et al. 2011; Wu et al. 2005; Qi et al. 2006), there is no established sensitive method regarding of simultaneous determination of astragaloside IV, cycloastragenol, calycosin-7-*O*-β-d-glucoside, calycosin, ononin and formononetin in seedlings was published.

Therefore, this study aimed at developing a sensitive and validated ultra-pressure liquid chromatography-electrospray ionization-mass spectrometry (UPLC-ESI–MS) method with a short time for simultaneous determination of astragaloside IV, cycloastragenol, calycosin-7-*O*-β-d-glucoside, calycosin, ononin and formononetin in *A. mongholicus* under different levels of salt stress.

## Methods

### Materials and reagents

Seeds of *A. mongholicus* were purchased from AnGuo Chinese Herbal Medicine Base in HeBei Province of China. Astragaloside IV, cycloastragenol, calycosin-7-*O*-β-d-glucoside, calycosin, ononin, formononetin, scutellarin (IS, internal standard) and sodium chloride were purchased from Chengdu Must Bio-technology Co., LTD. (Chengdu, China). Sodium chloride (NaCl) was of analytical reagent grade. Water used for the UPLC–MS/MS analysis was prepared with a Milli-Q water purification system procured from Millipore (Milford, MA, USA). Acetonitrile (J & K Scientific Ltd. Beijing, China) was of HPLC grade. All other chemicals used in the method were of analytical grade.

### UPLC–MS analysis

Chromatographic separation was performed on an Ultra-Performance LC (UPLC) system (Waters, Japan) with a LC-20AD pump, a temperature controller, column oven, SIL-20A autosampler (Waters, Japan), and ACQUITY UPLC BEH C18 Column (1.7 µm, 2.1 mm × 50 mm) with in-line filter and maintained at 25 °C was used. The mobile phase in UPLC determinations is consisted of (A) water and (B) acetonitrile. The elution program was optimized as follows: 8 % B (0–1 min), 8–34 % B (1–1.5 min), 34 % B (1.5–4 min), 34–60 % B (4–6 min), 60 % B (6–7 min), 60–8 % B (7–7.5 min), 8 % B (7.5–9 min). The flow rate was 0.25 mL/min and the injection volume was 5 μL.

Tandem mass spectrometric detection was performed on an QTRAP 5500 Ion trap mass spectrometer (AB SCIEX, USA) equipped with an electrospray ionization (ESI) source in the positive ion detection mode. MS source conditions were set as follows: Ionspray voltage: 5500 V, turbo spray temperature: 500 °C, high purity nitrogen was used in all units; nebulizer gas: 25 psi; curtain gas: 20 psi. Analytes were quantified by multiple reaction monitoring (MRM) mode. The optimized fragmentation transitions for MRM (precursor-to-product ion pair and parameters): *m/z* 806.9 ([M + Na]^+)^ → 627.1 ([M–C_6_H_11_O_5_–OH + Na]^+^) with declustering potential (DP): 117 V, collision energy (CE): 81 V and collision cell exit potential (CXP): 17 V for astragaloside IV; *m/z* 491.3 ([M + H]^+^) → 143.0 [M–C_22_H_36_O_3_ + H]^+^ with DP: 85 V, CE: 20 V and CXP: 12 V for cycloastragenol, *m/z* 446.9 ([M + H]^+^) → 284.2 ([M–C_6_H_11_O_5_ + H]^+^) with DP: 104 V, CE: 24 V and CXP: 8 V for calycosin-7-*O*-β-d-glucoside, *m/z* 285.0 ([M + H]^+^) → 269.9 ([M–CH_3_ + H]^+^) with DP: 117 V, CE: 33 V and CXP: 17 V for calycosin, *m/z* 431.0 ([M + H]^+^) → 268.2 ([M–C_6_H_11_O_5_ + H]^+^) with DP: 111 V, CE: 28 V and CXP: 15 V for ononin, *m/z* 269.0 ([M + H]^+^) → 196.2 ([M–OH–CO–OCH_3_ + H]^+^) with DP: 80 V, CE: 50 V and CXP: 17 V for formononetin, *m/z* 463.0 ([M + H]^+^) → 287.0 ([M–C_6_H_8_O_6_ + H]^+^) with DP: 100 V, CE: 29 V and CXP: 8 V for scutellarin (IS). The analysis of astragaloside IV achieved a positive ion of *m/z* 806.9, referring to the [M + Na]^+^ precursor ion. Data acquisition was performed with Analyst 1.4.2 software version (AB SCIEX, Concord, Ontario, Canada).

### Plant material, growth conditions and sample preparation

*Astragalus mongholicus* plants were grown in a greenhouse (ZPW-400, China), under a 14/10 light/dark photoperiod at a temperature of 25 (day)/22 °C (night) and 70 % relative humidity. Seeds of *A. mongholicus* were planted in pots using perlite as culture substrate and kept moistened until the seeds had germinated. 20 days seedlings were selected as initial material. Sodium chloride (NaCl) concentration in the culture substrate solution was adjusted to 0 (control), 100 and 300 mM. After 7 days, roots, stems and leaves under different salt levels were collected respectively.

The plant materials collected were pulverized to 5 μm by grinding instrument (MM 400, Retsch, GmbH, Haan, Germany), and 0.5 g tissue aliquots of roots, stems and leaves under different salt levels were extracted respectively. The plant samples were treated with 10 mL of 80 % ethanol (composed of ethanol and water in a volume ratio of 4:1, include 500 ng/mL IS), and reflux extraction was performed for 45 min. The samples extractions were filtered and the residues were treated with 10 mL of 80 % ethanol, and reflux extraction was performed for 45 min. The samples extractions were filtered and the filtrates were merged. Then the filtrates were dried under low pressure by vacuum cavitation instrument. The resultant extracted material was dissolved in mobile phase (1 mL) and filtered through micropores of 0.22 μm diameter. The purified solution was analyzed by UPLC-MS.

### Preparation of calibration standards and quality control (QC) samples

The standard stock solutions of astragaloside IV (1 mg/mL), cycloastragenol (1 mg/mL), calycosin-7-*O*-β-d-glucoside (1 mg/mL), calycosin (1 mg/mL), ononin (1 mg/mL) and formononetin (1 mg/mL) were prepared by dissolving required amount of the reference standards in methanol. A working solution of IS was prepared in methanol at a final concentration of 100 μg/mL. A mixed stock solution containing 1 mg/mL of the six reference standards and IS in methanol for chromatographic separation was prepared. The working standard solutions of calibration curve were prepared by successive dilution of the mixed stock solution with methanol to yield the final concentration series ranged from 1.48 to 14,800 ng/mL for astragaloside IV, calycosin-7-*O*-β-d-glucoside and ononin, and 0.148 to 1480 ng/mL for cycloastragenol, calycosin and formononetin. QC samples including 74 (low), 740 (mid) and 7400 ng/mL (high) for astragaloside IV, calycosin-7-*O*-β-d-glucoside and ononin, 7.4 (low), 74 (mid) and 740 ng/mL (high) for cycloastragenol, calycosin and formononetin were prepared as the same procedure as the calibration standards. Calibration work solutions and QC samples were kept at −20 °C until UPLC-MS/MS analysis. All of these were freshly prepared before each experiment.

### Method validation

The linearity of the method was generated by analysis of six calibration curves. The calibration standard curves of the tested compounds were obtained by least-squares linear regression of the peak area versus the concentrations, using the least-square linear regression with weighting factor 1/concentration^2^. All calibration curves were required to have a correlation value of at least 0.99. LLOQ was determined based on the signal-to-noise ratio of 10:1.

The intra- and inter-day precision and accuracy of the assay were determined by analyzing six replicates of three concentration levels of QC samples (low, mid and high concentrations) on the same day and on three consecutive days, respectively. Accuracy and precision were evaluated according to relative error (RE) and relative standard deviation (RSD), respectively. Values below ±15 % were accepted.

Extraction recoveries were evaluated at three QC levels (low, mid and high concentrations) by comparing the peak areas obtained from the samples with the analytes spiked before and after extraction. Matrix effect generally impacts in the form of either ion suppression or ion enhancement. The peak areas obtained from analytes in matrix extract (A) were then compared with corresponding peak areas obtained from the standard solutions in the mobile phase (B) at equivalent concentrations. The ratio (A/B × 100) is defined as the matrix effect. The value of matrix effect less than 85 % represented ionization suppression, while more than 115 % represented ionization enhancement.

Stability tests were performed for six replicates of QC samples at three concentrations (low, mid and high concentrations) under different conditions. The short-term and long-term stability was tested by analyzing samples exposed at room temp for 4 h and kept at −20 °C for 2 weeks, respectively. The freeze–thaw stability after three freeze–thaw cycles on three consecutive days was also determined. Stability was assessed by comparing the mean concentration of the samples with the mean concentration of freshly prepared calibration. The samples were considered stable if the assay values were within the acceptable limits of ±15 % RSD.

### Statistical analysis

Results were subjected to analysis of variance (ANOVA) to determine the significant differences between different levels of salt treatment times. If ANOVA was performed, Duncan’s honestly significant difference (HSD) post hoc tests were conducted to determine the differences between individual treatments (SPSS 17.0, SPSS Inc., USA).

## Results and discussions

### Optimization of UPLC-MS conditions

Because of the complexity of the plant components, many analogs maybe co-eluted during analyses. It is important to obtain a sensitive and accurate UPLC-MS method including efficient chromatographic separation and appropriate ionization. The chromatographic conditions were optimized simultaneously to reduce the influence of endogenous interferences, improve the peak shape, increase the signal response of analytes, and shorten the running time. Liquid chromatographic conditions were investigated such as column, mobile phase, column temperature and flow rate that could greatly influence the separation. In the present study, ACQUITY UPLC BEH C18 Column (1.7 µm, 2.1 mm × 50 mm) was chosen for its high efficiency and improving the peak shape. Different mobile phases (water–methanol, water-acetonitrile) were examined and compared. It was found that better peak shapes and elution efficiency could be produced by water-acetonitrile. It was also found that the best separation was obtained when the column temperature was kept at 26 °C using a flow rate of 0. 25 mL/min. Full scan positive ion ESI mass spectra were obtained for each of the compounds by direct injection of the standards of astragaloside IV, cycloastragenol, calycosin, ononin, formononetin, calycosin-7-*O*-β-d-glucoside and IS, respectively.

**Fig. 1:**
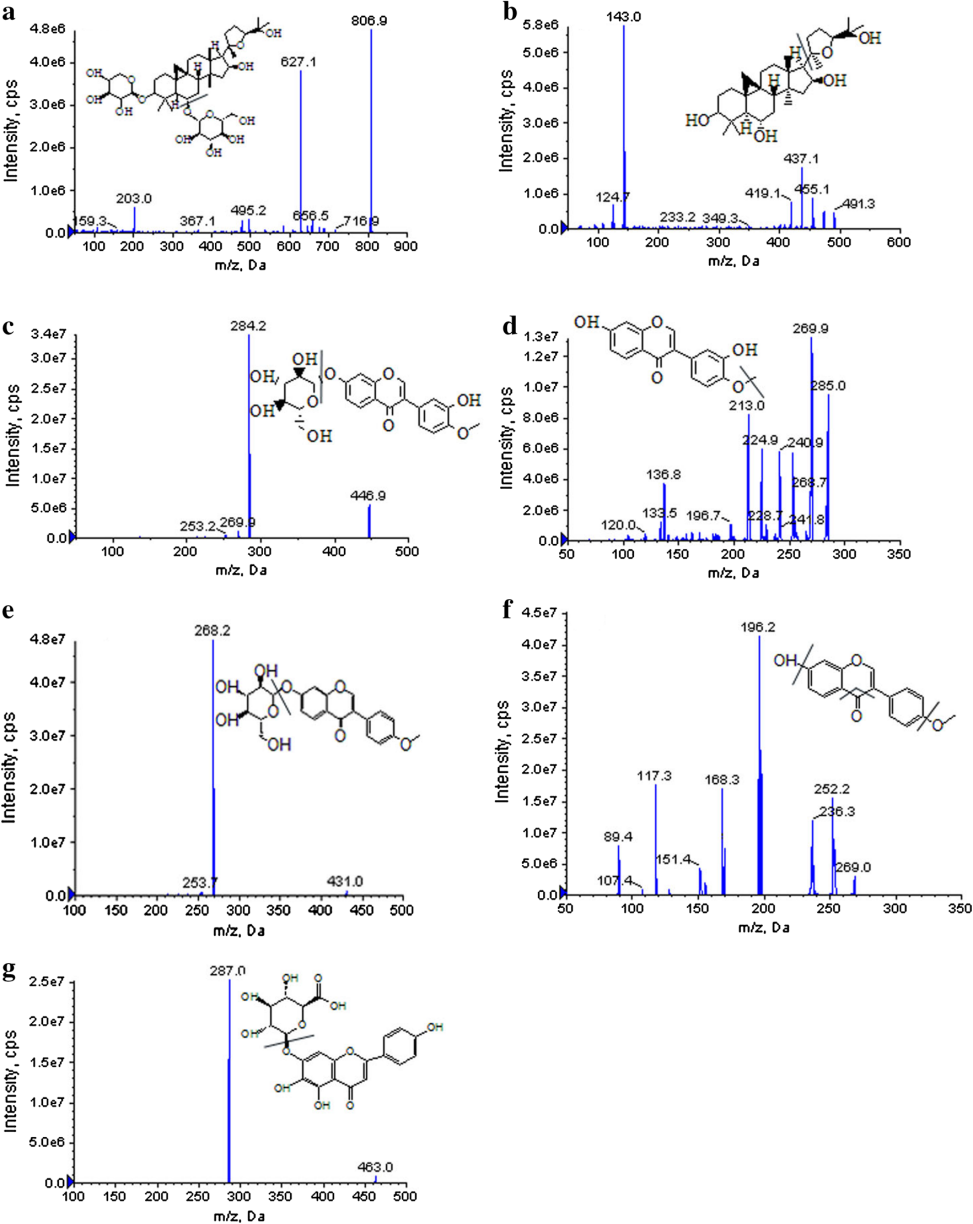
The production scan spectra and chemical structure of the tested compounds and scutellarin (IS): **a** astragaloside IV; **b** cycloastragenol; **c** calycosin-7-*O*-β-d-glucoside; **d** calycosin; **e** ononin; **f** formononetin; **g** IS

**Fig. 2:**
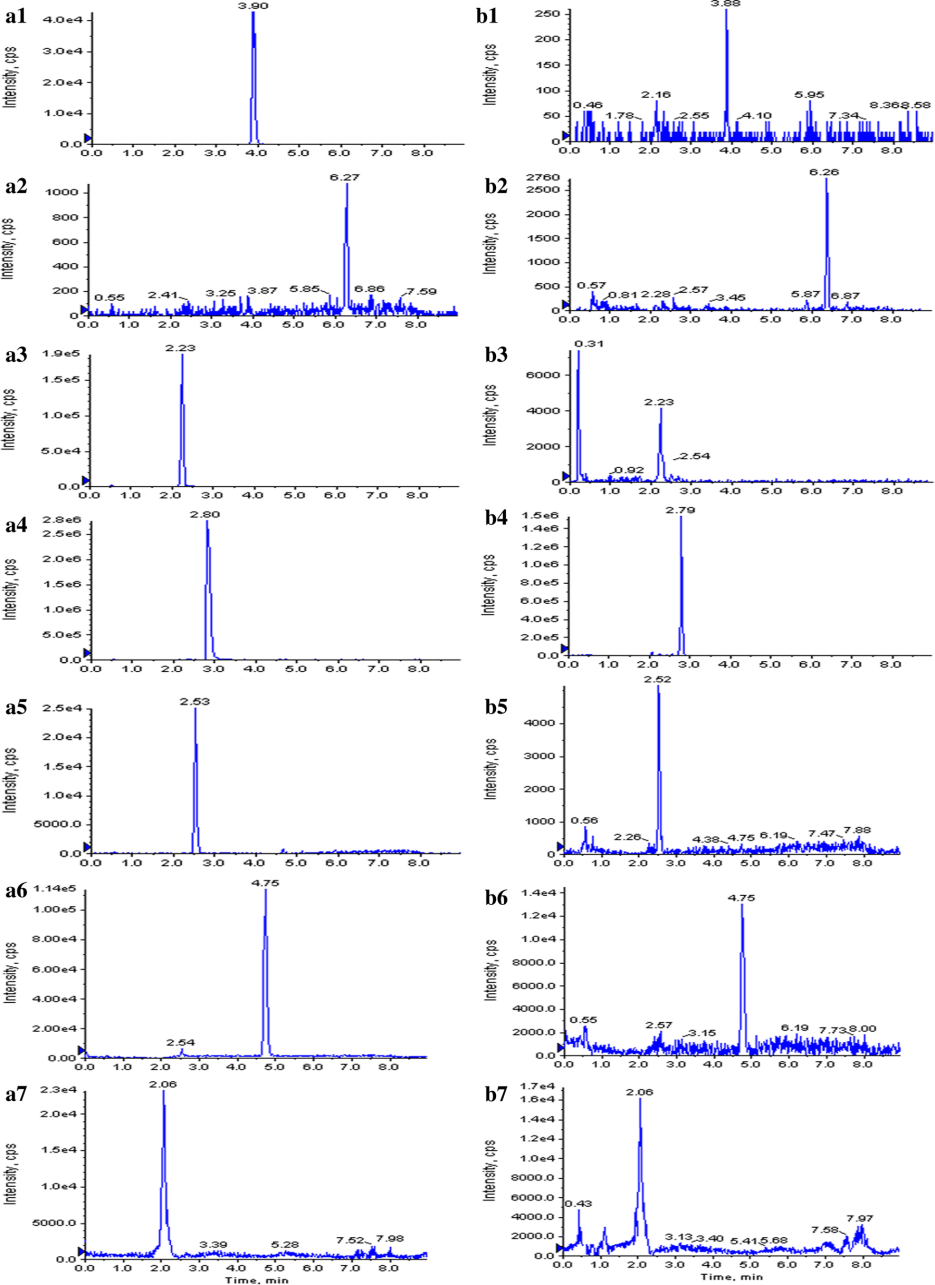
Representative MRM chromatograms of **a** six tested analytes and IS standard; **b** seed sample (*A1*, *B1*—astragaloside IV; *A2*, *B2*—cycloastragenol; *A3*, *B3*—calycosin-7-*O*-β-d-glucoside; *A4*, *B4*—calycosin; *A5*, *B5*—ononin; *A6*, *B6*—formononetin; *A7*, *B7*—scutellarin)

To optimize mass spectrometric conditions for detection of these compounds, several conditions were investigated such as scan mode, the precursor and product ions of these analytes, source temperature and ions spray voltage. Under ESI ion source condition, the response of these compounds observed in positive ion mode was higher than that in negative ion mode. Thus, positive ion mode was finally employed. The precursor and product ions of these analytes were ascertained by injecting standard solutions in Q1 scan and product ions (MS2) mode, respectively. In the precursor ion scan mass spectra, most analytes formed molecular ions [M + H]^+^, except that the ions of astragaloside IV were [M + Na]^+^. Astragaloside IV predominantly formed sodium adduct ions [M + Na]^+^, at *m/z* of 806.9 with the response higher than [M + H]^+^. However, both of these precursor ions formed the same fragment ion, with [M + H]^+^ being the most abundant. The [M + H]^+^ and [M + Na]^+^ ions were therefore chosen as the precursor ions to obtain their major fragment ions for MRM analysis. The fragment ions at *m/z* values of 627.1, 143.0, 284.2, 269.9, 268.2, 196.2 and 287.0 were present in the highest abundance for astragaloside IV, cycloastragenol, calycosin-7-*O*-β-d-glucoside, calycosin, ononin, formononetin and IS, respectively. The instrument was tuned to yield the maximum product ion for each compound. The MS/MS product ion spectra of these analytes are shown in Fig. 1. Then, the MS parameters, including ionspray voltage, decluster potential (DP), collision energy (CE) and collision cell injection potential (CXP), were optimized through the product ion mode. The optimized MS/MS transitions and energy parameters of these analytes were shown in Table 1. In order to obtain the maximum abundance of the molecular ions of the compounds, the source temperature, the nebulizing gas (N_2_) pressure and the curtain gas flow were set at 500 °C, 25 and 20 psi, respectively. A typical MRM chromatogram of the standards and the sample is shown in Fig. 2. The retention times of astragaloside IV, cycloastragenol, calycosin-7-*O*-β-d-glucoside, calycosin, ononin, formononetin and IS were 3.90, 6.27, 2.23, 2.80, 2.53, 4.75 and 2.06 min, respectively.

**Table 1:** The optimized MS/MS transitions and energy parameters of analytes

Analyte	DP (V)	CE (V)	CXP (V)	Precursor (amu)	Product (amu)
Astragaloside IV	117	81	17	806.9 [M + Na]^+^	627.1 [M–C_6_H_11_O_5_–OH + Na]^+^
cycloastragenol	85	20	12	491.3 [M + H]^+^	143.0 [M–C_22_H_36_O_3_ + H]^+^
Calycosin-7-*O*-β-d-glucoside	104	24	8	446.9 [M + H]^+^	284.2 [M–C_6_H_11_O_5_ + H]^+^
Calycosin	117	33	17	285.0 [M + H]^+^	269.9 [M–CH_3_ + H]^+^
Ononin	111	28	15	431.0 [M + H]^+^	268.2 [M–C_6_H_11_O_5_ + H]^+^
Formononetin	80	50	17	269.0 [M + H]^+^	196.2 [M–OH–CO–OCH_3_ + H]^+^
Scutellarin	100	29	8	463.0 [M + H]^+^	287.0 [M–C_6_H_8_O_6_ + H]^+^

### Method validation

The typical equations of calibration curves, the linearity ranges, correlation coefficients and LLOQs for the six analytes are shown in Table 2. Calibration data for each analyte were obtained using the optimized UPLC-MS method. The response profile was determined and observed to be linear for each of the compounds within the linear ranges. All calibration curves exhibited good linearity with correlation coefficient (r) within the range of 0.999–0.9999. It was observed that the LLOQs were 1.25 ng/mL for astragaloside IV, calycosin-7-*O*-β-d-glucoside and ononin, 0.125 ng/mL for cycloastragenol, calycosin and formononetin. These LLOQs are sufficient for content detection of the six compounds.

**Table 2:** Regression equations, linear ranges, correlation coefficients, and LLOQ of the tested compounds

Analyte	Linear range (ng/mL)	Calibration curves	r^2^ (n = 6)	LLOQ (ng/mL)
Astragaloside IV	1.48–14,800	y = 0.4672x + 0.00004	0.9995	1.25
Cycloastragenol	0.148–1480	y = 0.1243x + 0.00002	0.9998	0.125
Calycosin-7-*O*-β-d-glucoside	1.48–14,800	y = 0.7575x + 0.0017	0.9999	1.25
Calycosin	0.148–1480	y = 4.836x + 0.038	0.9992	0.125
Ononin	1.48–14,800	y = 0.1166x + 0.0001	0.9994	1.25
Formononetin	0.148–1480	y = 3.0566x + 0.0042	0.9995	0.125

The intra- and inter-day precisions and accuracies of QC samples are presented in Table 3. The intra- and inter-day precisions (RSD) of these analytes were all less than 2.33 and 4.16 %, while the accuracy was within ±6.87 % for all the analytes. The values for accuracy and precision demonstrated that the method is reliable and reproducible.

**Table 3:** The intra-day and inter-day accuracies and precisions of the tested analytes (n = 6)

Analyte	Nominal concentration (ng/mL)	Intra-day(n = 6)			Inter-day (n = 6)		
		Observed concentration (ng/mL)	Precision (RSD%)	Accuracy (RE%)	Observed concentration (ng/mL)	Precision (RSD%)	Accuracy (RE%)
Astragaloside IV	74	73.1 ± 0.11	1.46	−1.22	73.8 ± 0.14	3.25	−0.25
	740	737 ± 1.23	1.64	−0.47	746 ± 1.73	2.28	0.79
	7400	7395 ± 13.2	1.78	0.34	7505 ± 31.2	4.16	4.24
Cycloastragenol	7.4	7.39 ± 0.11	1.43	0.52	7.47 ± 0.21	2.85	4.35
	74	73.7 ± 1.21	1.64	−0.47	73.1 ± 1.31	1.72	−1.22
	740	742.8 ± 6.63	0.89	0.79	726.3 ± 2.43	3.24	0.88
Calycosin-7-*O*-β-d-glucoside	74	73.7 ± 1.29	1.64	0.89	73.1 ± 1.3	1.72	−3.18
	740	742.8 ± 6.67	0.89	0.79	726.3 ± 2.36	3.24	0.88
	7400	7383 ± 14. 7	0.67	−0.89	7393 ± 18.18	2.46	0.59
Calycosin	7.4	7.40 ± 0.17	2.33	−0.02	7.26 ± 0.15	2.01	−1.89
	74	74.37 ± 0.79	1.06	0.90	72.63 ± 2.48	3.41	0.88
	740	740.83 ± 5.85	0.79	−0.56	738.50 ± 15.15	2.05	0.48
Ononin	74	73.1 ± 0.13	1.72	−0.88	74.4 ± 0.15	2.06	6.87
	740	737 ± 1.2	1.64	−0.87	728 ± 1.7	2.40	−1.67
	7400	7440 ± 6.7	0.90	0.95	7363 ± 2.01	2.73	2.27
Formononetin	7.4	7.37 ± 0.09	1.27	0.29	7.49 ± 0.21	2.81	4.59
	74	73.7 ± 0.12	1.64	−1.14	73.4 ± 1.2	1.64	−0.77
	740	741.3 ± 5.6	0.76	0.59	731.3 ± 1.97	2.70	1.57

The average extraction recoveries of the QC samples are presented in Table 4. Mean recoveries of astragaloside IV, cycloastragenol, calycosin-7-*O*-β-d-glucoside, calycosin, ononin, and formononetin were 96.46–99.64, 98.21–100.54, 99.2–99.33, 93.63–98.63, 98.64–99.57, and 95.39–97.55 % at three QC levels. The data indicated that the extraction recoveries of these analytes were efficient, consistent and reproducible. The mean matrix effect values of astragaloside IV, cycloastragenol, calycosin-7-*O*-β-d-glucoside, calycosin, ononin, and formononetin were 99.65–99.81, 99.96–102.70, 97.71–101.50, 95.33–98.89, 97.47–101.47, and 99.65–104.36 %, respectively. In addition, the RSD showed that there was no severe variation across all QC concentration levels. These results indicated that these analytes did not exhibit obvious matrix effect under this UPLC-MS condition.

**Table 4:** Mean extraction recoveries and matrix effects of the tested analytes (n = 6)

Analyte	Spiked concentration (ng/mL)	Recovery (%)		Matrix effect (%)	
		Mean ± SD	RSD	Mean ± SD	RSD
Astragaloside IV	74	99.64 ± 0.002	0.28	99.81 ± 0.004	0.49
	740	99.61 ± 0.004	0.49	99.65 ± 0.012	1.27
	7400	96.46 ± 0.066	6.91	99.71 ± 0.024	2.41
Cycloastragenol	7.4	100.54 ± 0.031	3.17	102.70 ± 0.09	8.80
	74	98.21 ± 0.058	6.00	100.74 ± 0.063	6.25
	740	100.13 ± 0.004	0.49	99.96 ± 0.011	1.18
Calycosin-7-*O*-β-d-glucoside	74	99.20 ± 0.016	1.68	97.71 ± 0.05	5.20
	740	99.21 ± 0.01	1.10	98.53 ± 0.022	2.27
	7400	99.33 ± 0.015	1.54	101.50 ± 0.028	2.76
Calycosin	7.4	98.63 ± 0.026	2.71	98.89 ± 0.018	1.88
	74	97.17 ± 0.063	6.50	98.48 ± 0.042	4.31
	740	93.63 ± 0.081	8.73	95.33 ± 0.111	11.64
Ononin	74	99.57 ± 0.006	0.69	100.69 ± 0.013	1.36
	740	98.95 ± 0.017	1.77	101.47 ± 0.024	2.46
	7400	98.64 ± 0.021	2.17	97.47 ± 0.052	5.37
Formononetin	7.4	95.49 ± 0.07	7.36	99.65 ± 0.059	5.97
	74	97.55 ± 0.039	4.01	100.44 ± 0.007	0.78
	740	95.39 ± 0.081	8.59	104.36 ± 0.068	6.57

The results of stability experiments were summarized in Table 5 and it’s indicate that these analytes were stable in the QC sample at room temp for 4 h, −20 °C for 2 weeks and three freeze–thaw cycles. the RSD were found between 0.35 and 2.05 % for astragaloside IV, between 0.59 and 4.07 % for cycloastragenol, between 0.68 and 2.75 % for calycosin-7-*O*-β-d-glucoside, between 0.9 and 3.79 % for calycosin, between 0.92 and 3.11 % for ononin, and between 0.38 and 1.47 % for formononetin. No significant degradation of these analytes was observed during the 4 h storage at room temp, −20 °C for 2 weeks and the three freeze–thaw cycles. The responses varied from 0.35 to 4.07 % at all tested concentrations.

**Table 5:** The stability of the tested analytes (n = 6)

Analyte	Nominal concentration (ng/mL)	Short-term	Freeze–thaw cycles	Long-term
		25 °C for 4 h (RSD%)	Three freeze–thaw cycles (RSD%)	Frozen for 2 weeks (RSD%)
Astragaloside IV	74	0.35	1.21	2.05
	740	0.47	1.17	0.89
	7400	0.96	0.74	1.15
Cycloastragenol	7.4	0.59	2.1	0.78
	74	1.47	0.99	1.04
	740	0.85	4.07	1.54
Calycosin-7-*O*-β-d-glucoside	74	0.76	1.59	2.19
	740	0.97	2.75	0.68
	7400	2.41	1.34	1.3
Calycosin	7.4	1.02	3.52	2.11
	74	0.9	1.57	2.54
	740	1.53	3.79	1.15
Ononin	74	1.2	1.45	0.93
	740	0.92	2.97	1.06
	7400	2.36	3.11	2.35
Formononetin	7.4	1.14	0.82	1.47
	74	0.88	1.09	0.38
	740	0.56	1.18	1.02

### Accumulation of active metabolites in different organs under salt stress

**Fig. 3:**
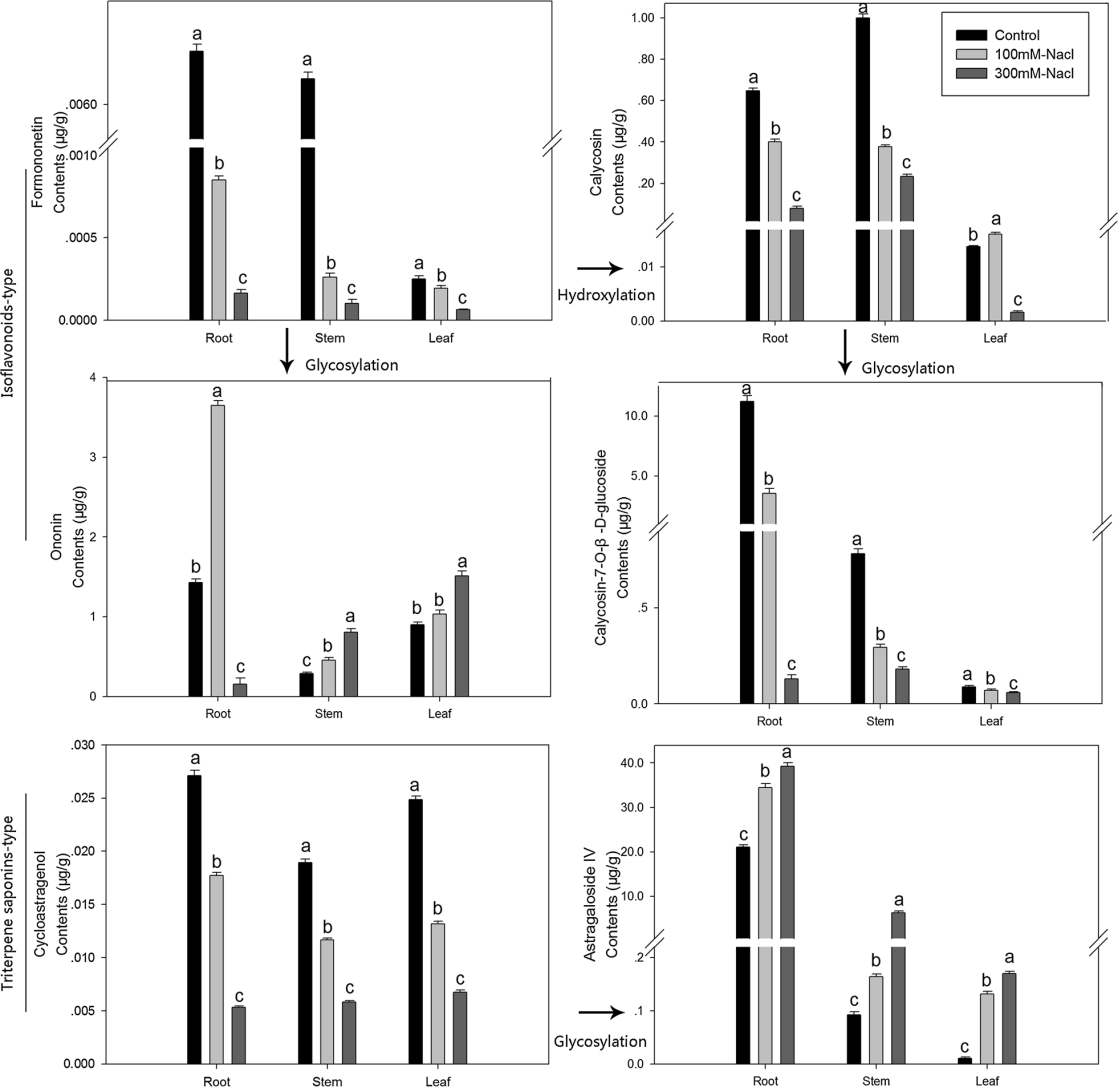
Content of six active metabolites in different organs of *A. mongholicus* under different salinity levels. Mean values with the same letter in the *single columns* were not significantly different according to Duncan’s multiple comparison test with a family error rate of 0.05

In the present study, the established UPLC-MS method has been successfully applied for the quantitative analysis studies of six bioactive compounds in *A. mongholicus* samples. Cycloastragenol and astragaloside IV are triterpene saponins; formononetin, ononin, calycosin and calycosin-7-*O*-β-d-glucoside are the major isoflavonoids in *A. mongholicus.* Representative MRM chromatograms of analytes standard (A) and samples (B) have shown in Fig. 2. Content of target compounds in root, stem and leaf under salt stress of different levels have shown in Table 6. Accumulation of target compounds in seedlings grown under control showed tissue specificity, Calycosin-7-*O*-β-d-glucoside, ononin, formononetin, astragaloside IV and cycloastragenol all have the highest content in root, which were 11,189.37, 1429.63, 6.78, 21,022.63 and 27.10 ng/g for fresh weight (Plant samples be excised and weighed immediately). Calycosin have the highest concentration in stem, which was 646.46 ng/g for fresh weight. Because of the numerous accumulation of the major bioactive metabolites in root, the root of *A. mongholicus* always been used as medicinal material. Significant variations in the content of 6 bioactive metabolites were observed after been processed by different levels of salinity in different part of plant (Fig. 3).

**Table 6:** Content of six active metabolites in different organs of *A. mongholicus* under different salinity levels

NaCl (mM)	Organs	Content of six active metabolites (ng/g, FW)					
		Calycosin-7-*O*-β-d-Glc	Calycosin	Ononin	Formononetin	Astragaloside IV	Cycloastragenol
0	Root	11,189.37	646.4608	1429.63	6.775	21,022.63	27.10247
	Stem	783.368	999.7942	286.63	6.375	92.12638	18.92748
	Leaf	87.168	13.79417	899.63	0.25	10.62637	24.85248
100	Root	3549.368	401.4608	3649.63	0.8525	34,497.63	17.72748
	Stem	294.368	376.4608	455.63	0.26	163.8764	11.67747
	Leaf	70.668	16.1275	1029.63	0.1925	131.1264	13.15247
300	Root	130.368	80.1275	156.63	0.16375	39,247.63	5.302475
	Stem	181.368	233.1275	806.63	0.102	6297.626	5.827475
	Leaf	57.468	1.6275	1509.63	0.06275	169.3764	6.752475

In the triterpene saponins pathway, cycloastragenol is the synthetic precursor compound of astragaloside IV. Cycloastragenol is relatively uniformly distributed in roots, stems and leaves. As the main active ingredients in *A. mongholicus*, astragaloside IV has massive accumulation in roots. Under the action of salt stress, cycloastragenol accumulated in root, stem and leaves was decreased. This suggests the possibility of two aspects: one is the synthesis of cycloastragenol being inhibited by salt stress; the other is the cycloastragenol consumption because of its glycosylation process being promoted by salt stress; while, the accumulation of astragaloside IV was significantly increased in roots, stems and leaves. This might means that astragaloside IV could extenuate the salt damage for plant, and also reveal the possibility of cycloastragenol conversion to astragaloside IV via glycosylation under salt stress.

In the isoflavonoids pathway, calycosin is the synthetic precursor compound of Calycosin-7-*O*-β-d-glucoside, formononetin is the common synthetic precursor of calycosin and ononin. As the main active ingredients in *A. mongholicus*, calycosin-7-*O*-β-d-glucoside has massive accumulation in roots. The content of calycosin-7-*O*-β-d-glucoside and its synthetic precursors calycosin and formononetin were declined with salinity in root, stem and leaves. This might be the conversion from formononetin to calycosin being inhibited, and leading to decline of calycosin and calycosin-7-*O*-β-d-glucoside contents. Meanwhile, ononin, synthesized by formononetin via glycosylation, showed contents increment in roots, stems and leaves under salt stress. This reveals that conversion of formononetin to ononin via glycosylation process was promoted; while its hydroxylation process to calycosin was inhibited, decreasing the synthesis of calycosin and calycosin-7-*O*-β-d-glucoside. A probable mechanism was prompted, glycosylation process of flavonoids pathway was promoted under salt stress. Ononin, a compound of flavonoids and phenolics in *A. mongholicus,* was synthesized in large quantities by formononetin via glycosylation to play roles in adapt salt ambience. Flavonoids usually accumulate transiently, as a plastic response to biotic or abiotic stressors (Del Valle et al. 2015). Flavonoids suppress ROS (reactive oxygen species) levels in guard cells and thereby modulate the dynamics of stomatal aperture (Watkins et al. 2014). Plant phenolics, like other natural compounds, provide the plant with specific adaptations to changing environmental conditions and, therefore, they are essential for plant defense mechanisms (Caretto et al. 2015).

The results presented the response of major active metabolites in different organs of *A. mongholicus* to different salinity levels, and provided support for further exploration of the salt-tolerant mechanisms in *A. mongholicus* and its possibility of being the species that control the soil salinization.

## Conclusions

A sensitive UPLC-MS method for the determination of astragaloside IV, cycloastragenol, calycosin-7-*O*-β-d-glucoside, calycosin, ononin and formononetin was developed, validated, and applied for their quantitative analysis in *A. mongholicus*. The described UPLC-MS method was sensitive, with high accuracy and a short run time of 9 min, and met all the requirements for effective plant extract analysis. With no reports on UPLC-MS methods for the quantitative determination of these six compounds, this is the first report of the simultaneous quantitative study of Astragaloside IV, cycloastragenol, calycosin-7-*O*-β-d-glucoside, calycosin, ononin and formononetin in different organs of *A. mongholicus* under different levels of salinity. The results provided basis and support for further exploration of the potential salt-tolerant species development. The method presented is valuable for providing a procedure for the future analysis of trace components in seedlings of *A. mongholicus* under salt stress.
